# More Success With the Optimal Motivational Pattern? A Prospective Longitudinal Study of Young Athletes in Individual Sports

**DOI:** 10.3389/fpsyg.2020.606272

**Published:** 2021-01-20

**Authors:** Michael J. Schmid, Bryan Charbonnet, Achim Conzelmann, Claudia Zuber

**Affiliations:** Institute of Sport Science, University of Bern, Bern, Switzerland

**Keywords:** person-oriented approach, motivation, pattern analysis, predicting success, individual sports

## Abstract

It is widely recognized that motivation is an important determinant for a successful sports career. Specific patterns of motivational constructs have recently demonstrated promising associations with future success in team sports like football and ice hockey. The present study scrutinizes whether those patterns also exist in individual sports and whether they are able to predict future performance levels. A sample of 155 young individual athletes completed questionnaires assessing achievement goal orientations, achievement motives, and self-determination at t_1_. The person-oriented method linking of clusters after removal of a residue (LICUR) was used to form clusters based on these motivational constructs in order to analyze the relations between these clusters and the performance level 2.5 years later (t_2_). Similar to the studies in team sports, four motivational patterns were observed at t_1_. The *highly intrinsically achievement-oriented athletes* were much more likely to compete internationally [odds ratio (*OR*) = 2.12], compared to the *failure-fearing athletes* (*OR* = 0.29). Although team and individual sports differ in many respects, they nevertheless are characterized by similar and thus generalizable career-promoting motivational profiles: Regardless of the type of sport, the *highly intrinsically achievement-oriented* athletes consistently have the best potential for success.

## Introduction

Current empirical research on motivation in sport has examined a broad range of theoretical constructs, including achievement motivation (e.g., Coetzee et al., 2006; Elbe and Beckmann, 2006; Sagar et al., 2010), achievement goal orientations (e.g., van Yperen and Duda, 1999; Reilly et al., 2000; Domínguez-Escribano et al., 2017), or self-determination (e.g., Sarrazin et al., 2002; Gillet et al., 2009, 2012), and highlighted their importance for sports performance. However, most of these studies systematically considered the different motivational variables in isolation. The failure to take the multidimensional nature of the motivational subsystem into account, results in neglect of compensation mechanisms as well as interactions between different variables, which are assumed by talent research (e.g., Bartmus et al., 1987). Thus, to offer tailored and person-oriented psychological assistance to youth elite athletes, it seems essential to understand the emerging dynamics within the motivational subsystem, and to examine the developmental consequences (e.g., prognostic value) of different combinations of motivational variables (or so-called patterns). Therefore, the use of a holistic and dynamic-interactionistic research paradigm seems to be appropriate (Bergman et al., 2003; Bergman and Andersson, 2010). One possibility is the application of a person-oriented approach, which provides a view of the system as a whole with its components forming a pattern that is considered indivisible (Bergman and Wångby, 2014). This pattern has to be understood and analyzed in its entirety and cannot be broken down into independent variables (Bergman and Wångby, 2014). So far, only three studies combined different motivational constructs with a person-oriented approach and the goal of identifying predictive patterns of future athletic performance (Zuber et al., 2015, forthcoming; Zuber and Conzelmann, 2019b). Zuber et al. (2015) used the Linking of Clusters after removal of a Residue (LICUR) method to form motivational patterns out of achievement goal orientations (i.e., win orientation and goal orientation), achievement motives (i.e., hope for success and fear of failure), and self-determination in young talented football players. Those patterns were relatively stable over the span of 1 year in early adolescents and effectively predicted future success in football after 1 (Zuber et al., 2015) and 5 years, respectively (Zuber et al., forthcoming). It was found that the *highly intrinsically achievement-oriented players* were significantly more likely to end up in the highest performance level after 5 years (*OR* = 3.5; Zuber et al., forthcoming). Those athletes showed high win and goal orientations, hope for success, and self-determination but low fear of failure. Conversely, the *non-achievement-oriented failure-fearing players* showed the opposite pattern and were, as a result, significantly less likely to compete at the highest performance level (*OR* = 0.4; Zuber et al., forthcoming). In young ice-hockey athletes, structurally similar motivational patterns demonstrated great relation with success over 6 months (Zuber and Conzelmann, 2019b).

These results suggest that, despite the differences related to the inherent specificity of these team sports, a promising motivational pattern labeled *highly intrinsically achievement-oriented players* seems to emerge on a recurrent basis. The pattern’s repeated association with a higher probability of future success not only makes it worth striving for, but also supports the possible generalization of its joined benefits beyond team sports. Because person-oriented studies were only conducted in male football and ice hockey until now, the extent of their generalizability is underexplored. Investigation of the applicability of this approach in national talent development programs will require the thorough examination of their generalizability to a more diverse range of environments, such as individual sports and athletes of both sexes (e.g., Johnston et al., 2018). With regard to the generalizability from team to individual sports, several empirical findings suggest differences in terms of motivational processes (e.g., Hollembeak and Amorose, 2005; Hanrahan and Cerin, 2009; Jonker et al., 2010; van de Pol and Kavussanu, 2012). The higher controllability and responsibility of the behavior in individual sports may change the optimal motivational pattern required to reach the highest performance level (e.g., Hanrahan and Cerin, 2009). For example, it was found that athletes competing in individual sports have higher ego orientation than those of team sports (Hanrahan and Cerin, 2009), as well as higher level of intrinsic motivation (Hollembeak and Amorose, 2005) and different self-regulatory skills (Jonker et al., 2010).

In order to identify career-promoting or impeding motivational profiles in individual sports, the present study aims to replicate the results that Zuber et al. (2015) found in team sports with a sample composed of athletes from individual sports. By doing so, not only the reproducibility but also the generalizability of the results will be investigated.

## Materials and Methods

### Participants

With the help of Swiss Olympic and several sports federations, 76 coaches were contacted, who in turn suggested 263 athletes to participate. With a response rate of 62.7%, the overall sample consisted of 165 young athletes from Switzerland. Complete data sets were available for a total of 155 athletes (60 females and 95 males) with a mean age of 16.47 years (*SD* = 2.21). These athletes were competing in badminton (*n* = 7), biathlon (*n* = 1), curling (*n* = 7), freestyle skiing (*n* = 3), golf (*n* = 7), judo (*n* = 15), artistic cycling (*n* = 3), track and field (*n* = 12), wheelchair athletics (*n* = 2), mountain biking (*n* = 5), sledding (*n* = 1), rowing (*n* = 65), swimming (*n* = 7), alpine skiing (*n* = 5), shooting (*n* = 9), tennis (*n* = 1), or equestrian vaulting (*n* = 5).

### Measures

As a replication study of Zuber et al. (2015, forthcoming) and Zuber and Conzelmann (2019b), identical measures were used to assess the different motivational characteristics of the athletes. The achievement goal orientations were assessed through the German version (Elbe, 2004) of the Sport Orientation Questionnaire (SOQ; Gill and Deeter, 1988). The two scales win orientation (e.g., “I have the most fun when I win”) and goal orientation (e.g., “I try hardest when I have a specific goal”) were used and displayed acceptable to good internal consistencies (*α*_WO_ = 0.84; α_GO_ = 0.77).

The achievement motivation was measured by using the German version of the short scale of the Achievement Motives Scale – Sport (AMS-Sport; Wenhold et al., 2009) with its two dimensions of hope for success (e.g., “I enjoy athletic tasks that are slightly difficult for me”) and fear of failure (e.g., “I am even afraid of failing at athletic challenges that I believe I can accomplish”). The internal consistencies were within a good range (*α_HS_* = 0.80 and *α_FF_* = 0.84).

The self-determination was determined with the Sport Motivation Scale (SMS; Burtscher et al., 2011). Similar to Vallerand (2001) and Zuber et al. (2015), the seven subscales of motivation (intrinsic motivation toward knowledge, accomplishment and stimulation, identified, introjected, external regulation, and amotivation) were combined to form a self-determination index. The scale displayed good internal consistencies with *α* = 0.83.

### Procedure

A longitudinal research design was applied to assess the motivational characteristics and the future athletic success of the participants. At the first measuring point (t_1_), the athletes were asked to complete the self-assessment questionnaires. Their initial performance level was assessed through the allocation of Swiss Olympic Cards (SOCs). The type of card assigned (none, regional, national, and international/elite) mainly reflects three aspects: the results in annual multidimensional tests carried out by the federations, the systematic estimation of each athlete’s potential carried out by their coach, and the achievement reached in competitions. At t_1_, the performance level of these athletes ranges from regional to international (i.e., competing at youth world championships), which corresponds to levels T1–T4 in the Foundations, Talent, Elite, and Mastery (FTEM) framework (Gulbin et al., 2013). Two and a half years later (t_2_), the performance levels of the athletes (1 = international level; 2 = national level and lower; and 3 = dropout) were identified through their results at national or international competitions. At t_2_, 50 athletes participated in international competitions, whereby several athletes were ranked on the podium at junior or U23 world championships and one athlete had won an Olympic medal. The remaining athletes either participated in national competitions (*n* = 83) or were no longer found in the result databases and had dropped out (*n* = 19).

Formal ethical approval was granted from the authors’ institutional review board before conducting the study. All athletes and their legal representatives (for athletes younger than 16 years) provided their written informed consent.

### Data Analysis

The LICUR method (see Bergman et al., 2003) was used to analyze the motivational subsystem. This person-oriented approach has already proven its usefulness in various talent studies, as the multi-dimensional nature of sports performance and athlete development seeks for holistic and dynamic-interactionist approaches (Zibung and Conzelmann, 2013; Sieghartsleitner et al., 2018). Within the person-oriented approach, “the individual is seen as an organized whole with elements operating together to achieve a functioning system where the involved elements interact in the process” (Bergman and Andersson, 2010, p. 157). Consequently, the interacting variables of a system are described as operating factors (Bergman et al., 2003).

The following statistical analyses were carried out according to the guidelines of Bergman et al. (2003). In a first step, the dataset was checked for outliers (residues), because such rarely occurring cases would otherwise distort the cluster solution. A threshold value of 0.8, measured by the squared average Euclidean distance computed on standardized variables, was chosen as distance to identify multivariate outliers. In a second step, a hierarchical cluster analysis (Ward method with average squared Euclidean distance) was performed. In order to optimize the solution, a partitioning cluster analysis (k-means method) was executed. The optimal cluster solution was selected through content aspects and the criteria formulated by Bergman et al. (2003). Only the operating factors with *z*-scores ≥ 0.5 were used to label the different clusters. In a third step, the transitions (developmental paths) between the clusters and performance levels were checked for significance using a Fisher’s exact test with a hypergeometric distribution (*p* < 0.05). By calculating the odds ratio (OR), the strength of association between clusters and performance level is quantified (*OR* = 1.0 as the expected value; OR < 1.0 means less and OR > 1.0 more transitions than expected by chance). Additionally, a one-way ANOVA was performed to test any cluster differences in age and years of training. Eta-square (*η*^2^) was used to estimate the effect size (0.01 = small, 0.06 = medium, and 0.14 = large; Cohen, 1988). The distributions of sex and initial performance level (i.e., SOC type) across clusters were also checked with a Fisher’s exact test. The described LICUR analysis was carried out with the statistics package ROPstat 2.0 (Vargha et al., 2015), all other analyses with IBM SPSS Statistics 26.0.

## Results

**Figure 1:**
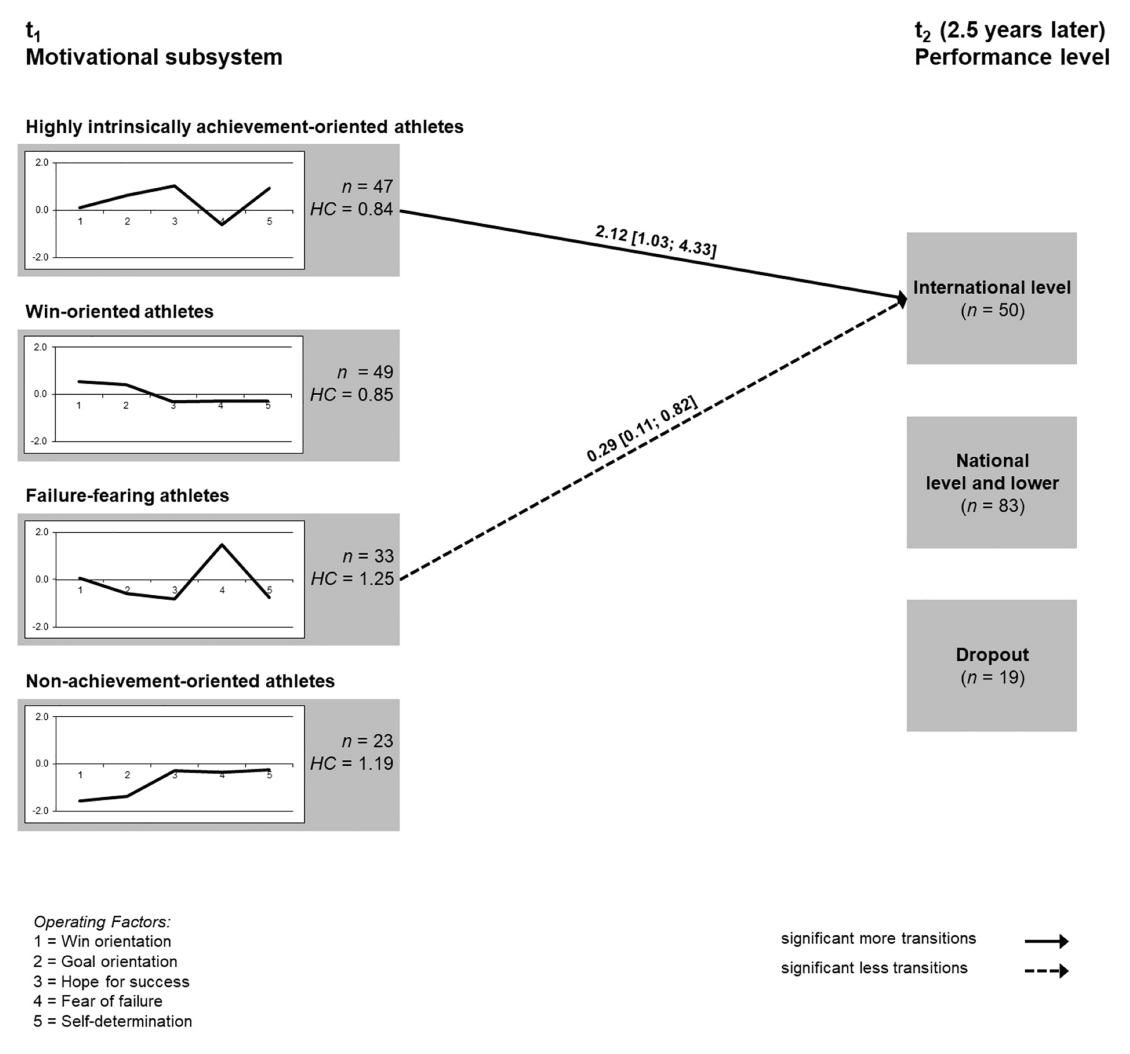
Profiles of *z*-scores of the four clusters and transitions to the performance levels at t_2_ (EESS = 51.78%). Operating factors: 1 = win orientation; 2 = goal orientation; 3 = hope for success; 4 = fear of failure; and 5 = self-determination index. Performance levels: 1 = international level; 2 = national level and lower; and 3 = dropout. HC, Homogeneity coefficient (mean square Euclidian distance within the cluster). The numbers next to the arrows represent the odds ratios (ORs) and 95% CIs (significant more transitions: OR > 1.0; significant less transitions: OR < 1.0).

Three cases exceeded the squared average Euclidean distance to all of the other cases and were therefore excluded from further analysis, resulting in a total sample size of 152 athletes. The descriptive statistics of the five motivational constructs before *z*-standardization are presented in Table 1. The four cluster solution (see Figure 1) was found to fit best in terms of content aspects as well as statistical criteria with an explained error sum of squares (EESS) of 51.78%, a weighted homogeneity coefficient (HC_mean_) of 0.99 (0.84; 1.25), and a silhouette coefficient (SC) of 0.58 (Vargha et al., 2016).

**Table 1:** Descriptive statistics for the subsystem motivation.

	Win orientation (range 1–5)		Goal orientation (range 1–5)		Hope for success (range 0–3)		Fear of failure (range 0–3)		Self-determination (range -18–18)	
	*M*	*SD*	*M*	*SD*	*M*	*SD*	*M*	*SD*	*M*	*SD*
Overall (*n* = 152)	3.97	0.75	4.49	0.47	2.23	0.49	0.56	0.59	7.82	3.24
Cluster 1 (*n* = 47) Highly intrinsically achievement-oriented athletes	4.14	0.68	4.72	0.30	2.78	0.21	0.14	0.22	9.94	2.74
Cluster 2 (*n* = 49) Win-oriented athletes	4.27	0.48	4.73	0.25	2.03	0.32	0.70	0.62	7.83	2.83
Cluster 3 (*n* = 33) Failure-fearing athletes	4.01	0.42	3.97	0.35	1.79	0.35	1.12	0.57	4.66	2.47
Cluster 4 (*n* = 23) Non-achievement-oriented athletes	2.67	0.58	3.92	0.41	2.13	0.39	0.40	0.27	7.07	2.61

Note that three cases have been classified as outliers and therefore were removed.

Cluster 1 (*highly intrinsically achievement-oriented athletes*) consists of athletes who display high goal orientation, hope for success, and self-determination, but low expression of fear of failure. Cluster 2 (*win-oriented athletes*) consisted of athletes with a particularly high win orientation. The athletes of the cluster 3 (*failure-fearing athletes*) are characterized by average values, apart from high fear of failure. Athletes with low win and goal orientation were grouped in cluster 4 (*non-achievement-oriented athletes*). There were no differences between the four clusters at t_1_ regarding age [*F*(3,148) = 0.22, *p* = 0.88, *η*^2^ < 0.01], years of training [*F*(3,148) = 1.36, *p* = 0.26, *η*^2^ = 0.03], and sex (*p* = 0.85). Results of the Fisher’s exact test revealed that the proportion of SOC types only differed in one cluster at t_1_. Specifically, in cluster 4 (*non-performance-oriented athletes*) athletes with a regional SOC were underrepresented (*p* = 0.01). Athletes of different sexes were evenly distributed across clusters (*p* = 0.85).

Two significant paths emerged from the clusters at t_1_ to the performance level t_2_. While the *highly intrinsically achievement-oriented athletes* were more likely to be found in the highest performance level at t_2_ [OR = 2.12 (1.03; 4.33)], the *failure-fearing athletes* were less likely [OR = 0.29 (0.11; 0.82)]. The two other clusters did not display any significant transitions to a specific performance level.

## Discussion

The purpose of the present study was to examine if the motivational patterns detected in the team sports football (Zuber et al., 2015) and ice-hockey (Zuber and Conzelmann, 2019b) can be observed in individual sports and demonstrate comparable developmental paths to a specific performance level. Overall, very similar but not identical clusters and developmental paths were discovered in the present study. It was shown that in individual sports, the *highly intrinsically achievement-oriented athletes* were more likely to reach the highest level. This cluster also displays many structural similarities to the most successful cluster in team sports (i.e., *highly intrinsically achievement-oriented players*). The three other clusters show slightly different patterns in comparison to the cluster solutions found in the previous team sport studies (cf. Zuber and Conzelmann, 2019b; Zuber et al., forthcoming). For example, no “average” cluster was identified. Athletes with the lowest prospects for future success in team sports were the *non-achievement-oriented failure-fearing players* (e.g., Zuber et al., 2015). This pattern did not emerge in individual sports; instead, the *failure-fearing athletes* displayed a significantly lower probability of competing on an international level at t_2_. In an equivalent manner to the team sport studies, the trend illustrated by this pattern accentuates the negative consequences of an above-average *fear of failure* and as a result, seems to point to the possibility of a generalizable career-limiting motivational profile.

Two patterns (*highly intrinsically achievement-oriented athletes* and *failure-fearing athletes*) and developmental paths emerge recursively, one positively and one negatively associated with future success. Regardless of the sport examined, there seem to exist career-promoting/−limiting motivational patterns for athletes.

From an applied perspective, the potential of an athlete’s person-oriented profiling and its implications in terms of coaching and selection must be critically reflected. Indeed, a careful evaluation of an athlete’s profile can help to design targeted psychological interventions, which in turn may help them to create their own career-promoting motivational pattern. For example, it has been shown that coaches can trigger higher self-determined motivation and sport performance in athletes by increasing autonomy support, which would be an interesting intervention for athletes with low self-determination (Gillet et al., 2010). Furthermore, cognitive-behavioral intervention designed to promote a mastery-initiating motivational climate was found to lower the trait anxiety among youth athletes (Smith et al., 2007). Thus, future studies have an opportunity to examine whether those motivational patterns can be altered over time through sport psychological interventions.

Even if such a questionnaire-based procedure seems recommendable from a developmental point of view and its prognostic value is good, it is highly problematic to use it in the selection context because social desirability would quickly bias the results (Zuber and Conzelmann, 2019a). Therefore, it is recommended not to use self-reporting questionnaires for talent identification but only for talent development. In order to consider achievement motivation in the talent identification and selection process, an external rating scale for the assessment of the achievement-motivated behavior could be used (Schmid et al., 2020; Zuber et al., 2020).

The restricted comparability of different sports included in this sample should be mentioned as a primary limitation of the study. Even if they are all individual sports, there are major differences between these sports. For example, golf as a precision sport, judo as a martial art, or swimming as an endurance sport pose quite different demands on a person (physical capabilities, mental skills, etc.). Indeed, it remains unclear whether an unfavorable configuration of motivational variables can be compensated more easily in certain sports, as the motivational variables might not have the same importance (e.g., Vaeyens et al., 2008). Future studies in this area should therefore examine the degree of probation of the career-limiting and/or career-enhancing motivational patterns by using extensive samples within certain sports. Nevertheless, in terms of generalizability, it would be interesting to test whether these motivational patterns could be found in a larger sample that includes a wider range of individual and team sports.

Moreover, because the importance of the motivational subsystem may vary in relation to other multidimensional factors characterizing talent during the time span of talent development (Baker et al., 2019), it would be of particular interest to examine the development of the prognostic value (e.g., ORs) of the different motivational patterns in a dynamical manner (e.g., early, middle, and late adolescence). Indeed, as noted by Ivarsson et al. (2020), potential moderators (e.g., age) might affect the strength of the relationship between psychological factors and future performance.

In conclusion, despite minor limitations, this study replicates the findings of previous studies regarding the application of motivational patterns for success prediction in young athletes (Zuber et al., 2015, forthcoming; Zuber and Conzelmann, 2019b). The general applicability of the method was demonstrated across multiple individual sports as well as athletes of both sexes, thus underlining the importance of considering motivational variables in talent development.

## Data Availability Statement

The raw and anonymized data supporting the conclusions of this article will be made available by the authors, without undue reservation, to any qualified researcher.

## Ethics Statement

The studies involving human participants were reviewed and approved by Ethics committee of the Phil.-hum. Faculty of the University of Bern. Written informed consent to participate in this study was provided by the participants’ legal guardian/next of kin.

## Author Contributions

MS, BC, AC, and CZ made significant contributions to the conception of the work, interpretation of the data, writing and revising the manuscript, and approved the submitted version. CZ and MS: data collection. MS: data analysis. All authors contributed to the article and approved the submitted version.

### Conflict of Interest

The authors declare that the research was conducted in the absence of any commercial or financial relationships that could be construed as a potential conflict of interest.
